# Does Childhood Diarrhea Influence Cognition Beyond the Diarrhea-Stunting Pathway?

**DOI:** 10.1371/journal.pone.0047908

**Published:** 2012-10-31

**Authors:** Christa L. Fischer Walker, Laura Lamberti, Linda Adair, Richard L. Guerrant, Andres G. Lescano, Reynaldo Martorell, Relana C. Pinkerton, Robert E. Black

**Affiliations:** 1 Department of International Health, Johns Hopkins Bloomberg School of Public Health, Baltimore, Maryland, United States of America; 2 Department of Nutrition, University of North Carolina, Chapel Hill, North Carolina, United States of America; 3 Center for Global Health, University of Virginia, Charlottesville, Virginia, United States of America; 4 Department of Parasitology, U.S. Naval Medical Research Unit No. 6 (NAMRU-6), Lima, Peru; 5 School of Public Health and Management, Universidad Peruana Cayetano Heredia, Lima, Peru; 6 Hubert Department of Global Health, Rollins School of Public Health, Emory University, Atlanta, Georgia, United States of America; Tulane University School of Public Health and Tropical Medicine, United States of America

## Abstract

**Background:**

Diarrhea is a leading cause of morbidity among children under 5 years of age in low- and middle-income countries yet the additional effects and sequelae, such as cognitive impairment associated with diarrhea, have not been quantified.

**Methods:**

We quantified the association between diarrhea prevalence and cognitive outcomes while controlling for linear growth in 4 study populations. Cognition was assessed using different methods across sites and was expressed in standardized units. We built linear regression models for each study with standardized cognitive score as the outcome and diarrhea prevalence as the main predictor variable. We then conducted meta-analyses of the regression coefficients to generate pooled estimates of the association between diarrhea prevalence and cognition whilst controlling for anthropometric status and other covariates.

**Results:**

Diarrhea was not a significant predictor of cognitive score in any site in the regression models or in the meta-analyses (Coefficient = 0.07; 95% CI: −0.1, 0.2). The length for age Z- score was negatively related to cognition in all sites (0.18; 95% CI: 0.14, 0.21), with coefficients remarkably similar across sites (Coefficient Range: 0.168–0.186).

**Conclusions:**

We did not demonstrate an association between diarrhea and cognition with stunting included in the model. The links between diarrhea, stunting, and cognition provide additional rationale for accelerating interventions to reduce diarrhea.

## Introduction

Diarrhea is a leading cause of morbidity and mortality among children under 5 years of age in low- and middle-income countries [1]. While the most severe diarrhea sequela, child mortality, is easily quantifiable, the indirect effects of diarrhea on subsequent risk of infection, growth, and cognitive ability are often less obvious and thus the full disease burden attributable to diarrhea is not as well understood [2].

Frequent diarrhea during the first 2 years of life has been negatively correlated with cognitive development and early school performance [3], [4], yet the pathway for this association is not fully understood. In an analysis of 9 data sets, Checkley at al estimated that each episode of diarrhea in early childhood increases the odds of being stunted at 24 mo of age by 5 [5]. In addition, it has also been shown that early childhood stunting contributes to cognitive impairment [6], [7], thus it is possible that diarrhea may be associated with a portion of cognitive impairment via the well-documented nutrition and infection cycle.

There is also evidence suggesting that cognitive delays may be related to differences in caregiver behavior toward a sickly child. During the acute and convalescent period of an illness, the child may be listless and less eager to play or explore his/her environment [8]. Children with prolonged or frequent episodes of diarrhea spend an increased proportion of days in the acute or recovery phase of illness and thus are likely to receive less stimulation by their own activity or by caregivers during these periods [8], [9].

We sought to quantify the association of diarrhea on cognitive outcomes while controlling for stunting in 4 datasets from 4 distinct study populations. Published studies have found an association between diarrhea and cognition, but often do not take into consideration anthropometric status [3], [4]. By including only datasets with individual level data on diarrhea prevalence, anthropometry at 2 years of age, and subsequent cognitive outcomes we are able to consider the relationship of diarrhea to cognition distinct from associations observed via the stunting pathway.

## Methods

### Selection of Data Sets

We conducted a PubMed search to identify possible studies for inclusion using the following search terms: *diarrhea, stunting, length-for-age, growth, cognitive impairment, cognition*, and *children*. We searched for studies that conducted routine diarrhea surveillance among populations of children under 2 years of age for at least 1 year, collected anthropometric indicators and conducted a follow-up of cognitive development at least 1 year after the initial diarrhea surveillance period. We excluded studies that did not use a published methodology for assessing cognition.

### Regression analysis

We sought to determine if diarrhea prevalence between 0 and 2 years of age had an impact on cognitive development later in life controlling for stunting at 2 years. To do this we built linear regression models for each study and subsequently conducted meta-analyses to pool each of the beta coefficients produced by these regressions. In the selected studies, cognitive scores were reported using 4 different methodologies and scales: non-verbal IQ [7], [10], Test of Non-verbal Intelligence III (TONI III) [11], Wechsler Intelligence Scale for Children-Revised [12], and the first factor of a factor analysis of eight tests representing general perceptual-organizational and verbal abilities [13] in Philippines, Brazil, Peru, and Guatemala, respectively (Table 1). To account for the use of different cognitive scales across study sites, we generated standardized scores by subtracting the mean and dividing by the standard deviation.

**Table 1:** Description of key study variables and variation in cognitive testing strategies across studies.

	Guatemala	Philippines	Brazil	Peru
**Sample size**	252	2117	111	143
**Sex (% boys)**	54.8	52.9	40.5	53
**Stunted (% <−2 LAZ)**	86.5	67.5	24.3	32
**Maternal education (mean years enrolled in school)** a ** or % of mothers who had not completed primary school** b	1.4^a^	7.0^a^	81.1%^b^	7.3^a^
**Cognitive Follow-up**				
**Age at cognitive test, ** ***mean (range) in years***	4 (±1 week)	8.5 (8.4–8.7)	8 (6–12)	9.4 (8.4–10.1)
**Type of cognitive test**	First factor score of eight tests	Non-verbal IQ	TONI IQ	Wechsler (WISC-R)
**Overview of cognitive test**	Perceptual-organizational and verbal abilities	Cognitive test designed to assess fluid ability (i.e. analytic or reasoning skills)	Non-verbal intelligence, aptitude, abstract reasoning and problem solving. Language free validation in non-English speaking groups.	Three separate IQs calculated from 10 subtests, a Verbal Scale IQ, a Performance Scale IQ, and a Full Scale IQ

a) Maternal education expressed as mean years enrolled in school.

b) Maternal education expressed as % of mothers who had not completed primary school.

For each study, we fitted two regression models with the standardized cognitive score as the outcome and diarrhea prevalence, defined as the percentage of days/study periods in which the caregiver reported diarrhea, as the main predictor variable. Model 1 controlled for length-for-age (LAZ) at age 2 as a continuous variable and model 2 controlled for LAZ as a categorical variable (with LAZ ≤−2 as a the cut off for stunted vs. not stunted). LAZ was defined by the World Health Organization reference standard [14]. In both models, we also controlled for variables commonly thought to influence cognitive function and those found to be significant in bivariate models: sex, mother's education, age at cognitive evaluation, and socioeconomic status (SES). With the exception of sex and LAZ, the remaining covariates were not treated uniformly across regression analyses. Variation in variable definitions could not be avoided because all studies were uniquely designed and not for the purpose of combining data. SES was defined as log household income and asset ownership in the Philippines; monthly income and the number of household rooms per person in Brazil; log household income in Peru; and multi-component household SES score (through factor analysis) and village residence in Guatemala. Age at cognitive evaluation was recorded in months and defined as a continuous variable in the Philippines, Brazil and Peru; in Guatemala, children were four years of age at cognitive assessment and thus categorical indicator variables for birth year were included in the model. In the Philippines, Peru and Guatemala, maternal education was defined as the average number of years enrolled in school; whereas, in Brazil, maternal education was a categorical variable representing the percentage of mothers that had not completed primary school. While the covariates included in each of the four regression models were not identical, all models controlled for each of these defined variables.

#### Meta-analysis

We conducted meta-analyses to pool regression beta coefficients for variables with exact definitions across all four regression models (i.e. diarrhea prevalence, LAZ, and sex). In order to generate pooled estimates of regression beta coefficients, we used Comprehensive Meta Analysis (CMA) software, which requires the coefficient and its standard error [15]. We used STATA 11 to determine the z-statistic corresponding to the p-value of each coefficient and then estimated the standard error by dividing the coefficient by its z-statistic [16]. We conducted random effects meta-analyses for each beta coefficient to account for both the within and between study variation that is expected with data from four different study sites. We also plotted a funnel plot to look for possible bias among the included studies.

## Results

We identified four studies that met our inclusion and exclusion criteria. The studies were conducted among 4 pediatric populations in low-income settings with ongoing diarrhea surveillance; details of the studies have been previously published [4], [13], [17], [18], [19], [20], [21]. In brief, children in these populations have high rates of infectious disease and are of low socioeconomic status. Diarrhea rates ranged from 4% of days in Brazil to 19% of days in The Philippines [4], [13], [17], [18], [19], [20], [21]. Table 1 describes the sample size and key study variables included in the analysis.

We conducted linear regression models for each study site to determine the within study effect, if any, of diarrhea on cognition. Diarrhea was not a significant predictor of cognitive score in any site (Table 2). The LAZ score coefficient was consistent across the 4 study populations and associated with cognition such that lower LAZ scores were related to lower cognitive performance (Coefficient Range: 0.168–0.186) in these multivariate analyses.

**Table 2:** Results of country-level linear regression models to determine the association of diarrhea on cognition.^a^^,^ b ^,^ c ^,^ d ^,^ e.

	Philippines			Brazil			Peru			Guatemala		
	Coefficient	SE	p value	Coefficient	SE	p value	Coefficient	SE	p value	Coefficient	SE	p value
**% days/periods with diarrhea**	0.158	0.130	0.225	−2.900	2.162	0.183	1.207	1.277	0.347	0.030	0.070	0.700
**LAZ** **(Continuous Variable)**	0.177	0.019	0.000	0.186	0.081	0.024	0.168	0.092	0.070	0.170	0.060	0.000
**Sex (0 = female, 1 = male)**	−0.084	0.040	0.034	−0.155	0.187	0.409	−0.318	0.195	0.106	−0.210	0.130	0.110

a . For each data set, standardized cognitive Z-score served as the outcome of linear regression.

b . All four regression analyses controlled for diarrhea prevalence, stunting and sex in the same way. Diarrhea prevalence was coded as the percentage of days/periods during which diarrhea was reported; LAZ was treated as a continuous z-score; sex was a categorical variable (0 = male, 1 = female).

c . SES was controlled for differently in each country given the data available from each site: 1) Philippines: controlled for log household income and ownership of assets; 2) Brazil: controlled for monthly income and the number of rooms in the household per person; 3) Peru: controlled for log household income; and 4) Guatemala: controlled for a multi-component SES score (through factor analysis) for the household as a continuous variable and residence in one of four villages.

d . Age (in months) at cognitive evaluation was treated as a continuous variable in Philippines, Brazil, and Peru.. In Guatemala, the children were 4 years of age at cognitive assessment, and therefore age was controlled for by the addition of categorical indicator variables for birth year to the regression model.

e . In Philippines, Peru and Guatemala, maternal education was defined as the average number of years enrolled in school. In Brazil, maternal education was a categorical variable representing the percentage of mothers that had not completed primary school.

**Figure 1:**
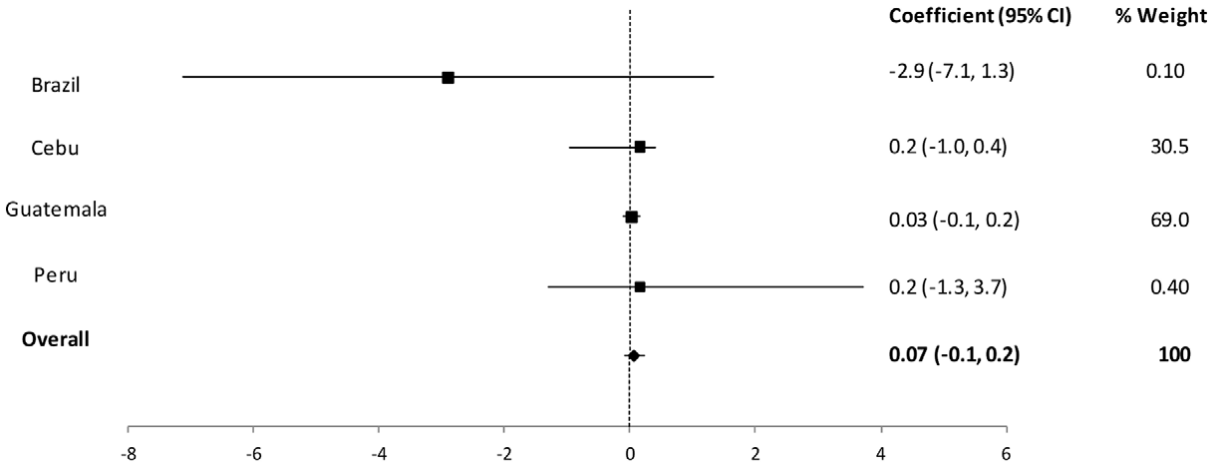
Forest plot of the change in cognitive Z-score per 1% increase in diarrhea prevalence controlling for LAZ score as a continuous variable, mother's education, sex, age at cognition and SES.

**Figure 2:**
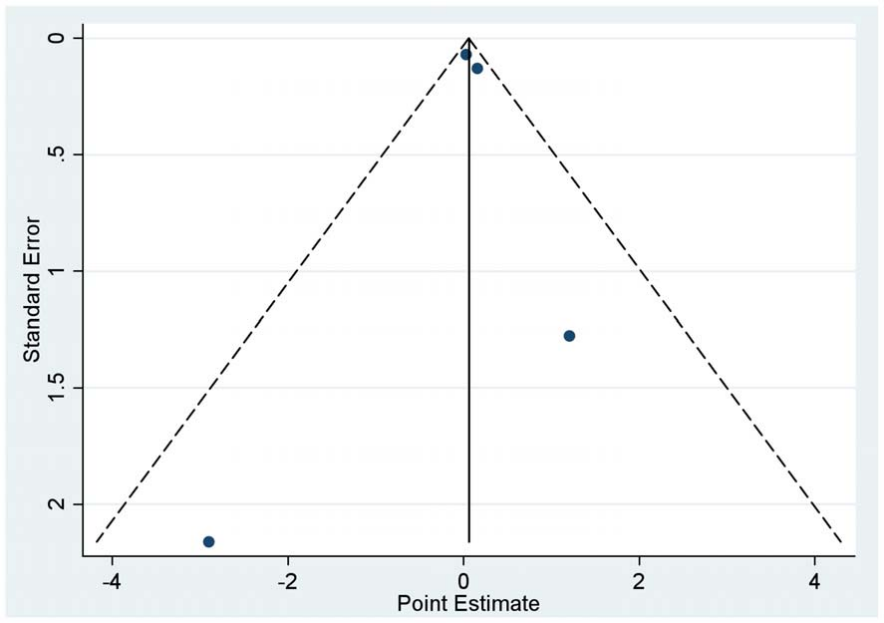
Funnel plot analysis of possible bias in the 4 studies included in the meta-analysis of the beta coefficient for diarrhea prevalence.

We then conducted meta-analyses to pool the regression coefficients across study sites and thus determine the cross-country effect of diarrhea on cognition whilst controlling for anthropometric status. The results of the two models were similar; here we present the pooled results of model 1 which considered length-for-age Z score as a continuous variable (Table 3). In pooled analyses, there was no statistically significant association between diarrhea and cognitive development (Coefficient = 0.07; 95% CI: −0.1, 0.2) (Figure 1). The symmetry observed in the funnel plot figure suggests that none of the included studies are biasing the results (Figure 2).

**Table 3:** Results Interpretation: Meta-analysis of the output from the regression of standardized cognitive z-score onto LAZ and diarrhea prevalence as continuous variables (model 1).^a^.

Coefficient	Pooled Coefficient from Random Effects MA(95% CI)	p-value	Interpretation of Coefficient
% days with diarrhea	0.07 (−0.09, 0.23)	0.39	Cognitive z-score increased by 0.0695 per 1% increase in days with diarrhea among children, holding all other variables constant
LAZ continuous	0.18 (0.14, 0.21)	<0.001	Cognitive z-score increased by 0.1764 per Z-score increase in length for age in among children, holding all other variables constant
Sex	−0.10 (−0.18, −0.03)	0.004	Cognitive z-score was 0.1048 lower among males compared to females, holding all other variables constant

a . We only report the pooled coefficients for variables with like definitions across study sites (i.e. diarrhea prevalence, LAZ, sex). Variables treated differently across study sites were not pooled (i.e. SES, age at cognitive assessment, maternal education).

## Discussion

We conducted a meta-analysis to assess the relationship between diarrhea and cognition while controlling for LAZ. We included 4 data sets from 4 different study populations and did not see an overall effect of diarrhea prevalence rates during the first 2 years of life on cognitive scores several years later. We did not observe an association of diarrhea prevalence and cognitive scores yet we did observe a remarkably consistent association of LAZ score and cognitive development across data sites. Thus, in these data sets a portion of cognitive impairment might be explained by the child's length deficit and this is independent of the child's diarrhea prevalence rate. It may be possible that diarrhea is a contributing factor to childhood stunting in these low-income settings but in this setting diarrhea prevalence alone did not appear to be associated with poorer cognitive performance in these children.

Some studies have observed associations between increasing diarrhea prevalence rates during early childhood and deficits or delays in later cognition [3], [4]. However, these studies have not taken into account the child's anthropometric status at 2 years. Checkley and colleagues found diarrhea prevalence to account for 20% of the stunting prevalence, and thus one might suspect that diarrhea may account for some portion of the stunting- cognition relationship [5].

Our analysis has several potential methodological weaknesses. The studies included here assessed cognition with 4 distinct methodologies. These studies were not designed with this meta-analysis in mind; rather, they were designed to use the best-standardized cognitive test available, which could be locally adapted to each setting. We accounted for the differences in scoring practices by using a statistically standardized score for each regression; this enabled us to include all study sites in the meta-analysis, even though the outcome variables were scaled differently. The studies included here also had variable follow-up time and did not have standard socio-economic covariates. Though we accounted for these differences analytically, the analysis would have been stronger if these covariates were standard across the datasets.

We included age in the regression model for all countries. Although cognitive test scores are age standardized, the standardization method differs among tests, and thus including this variable enabled us to control for residual confounding. In Philippines and Brazil, we observed a residual effect of age. In the Philippines, older children scored higher on the cognitive tests above and beyond what is accounted for in age-standardized test scores. However, in Brazil we observed a negative relationship between age at testing and cognitive performance. This may reflect increasing disparities over time such that children who demonstrate more advanced cognitive abilities early on and who live in stimulating environments continue to progress while children who have a lower skill level and are raised in non-stimulating environments fail to progress [22], [23], [24]. The gap between the two groups then widens as children age. In Brazil, diarrhea prevalence was found to be associated with lower cognitive test scores when the age variable was removed from the regression suggesting a correlation between age at testing and diarrhea prevalence (R. Pinkerton, personal communication). In Brazil this might be due in part to progressive declines in early childhood diarrhea burdens during a decade of surveillance [25]. It appears that the effect of age on cognition is context dependent because it varies among these four studies. Socioeconomic conditions may confound the putative relationship between diarrhea and impaired cognition because poor or less educated families are likely to have both high rates of childhood diarrhea and delayed careseeking [26], [27]. In impoverished households maternal and paternal education levels are often low and children receive less stimulation during times of health. It is likely that effects of diarrhea and/or stunting on cognition would be exacerbated in these deprived home environments during the diarrhea illness as stimulation would decline even further [28]. Studies have shown that the addition of stimulation provides an additional benefit to that of nutrition alone in improving mental development among stunted children [6]. Because cognitive function can be affected by both biological and environmental factors, studies that examine the possibility of confounding or interaction are the best to help us understand the possible role of diarrhea as a risk factor for cognitive impairment.

The complex pathways by which nutrition, infection, and the home environment affect a child's development are not well understood. Although there are limited data suggesting that numerous factors are at play, few studies have been able to look at all factors thus the roles of each remain elusive. Berkman et al observed that nutrition, parental education and school related factors independently affected cognition, but similar literature is scarce [19]. Our understanding of the complex relationships among diarrhea, nutritional status, poverty, careseeking, and developmental stimulation in the home would benefit greatly from additional studies explicitly designed to consider the role of each of these factors individually and more importantly to consider the mediating effects of one factor on another.

We did not demonstrate an association between diarrhea on cognition. Stunting remained a statistically significant covariate in this analysis showing a remarkably homogeneous association and suggesting a consistent relationship between stunting and cognition. While diarrhea may not directly impact cognitive impairment, the links between diarrhea and stunting, and stunting and cognition are clear and provide additional rationale for accelerating interventions to reduce diarrhea.
